# IFNL4 and IFNL3 Associated Polymorphisms Strongly Influence the Spontaneous IFN-Alpha Receptor-1 Expression in HCV-Infected Patients

**DOI:** 10.1371/journal.pone.0117397

**Published:** 2015-02-12

**Authors:** Licia Bordi, Claudia Caglioti, Anna Rosa Garbuglia, Daniele Lapa, Concetta Castilletti, Chiara Taibi, Maria Rosaria Capobianchi, Eleonora Lalle

**Affiliations:** 1 Laboratory of Virology, National Institute for Infectious Diseases “L. Spallanzani,” Rome, Italy; 2 Clinical Department, National Institute for Infectious Diseases “L. Spallanzani,” Rome, Italy; Harvard Medical School, UNITED STATES

## Abstract

Single-nucleotide polymorphism in IFNL3 gene (rs12979860) predicts spontaneous and therapy-induced HCV clearance. In a previous study from our group PBMC from patients with favourable rs12979860 genotype showed higher levels of IFNAR-1 mRNA. Recently, a dinucleotide polymorphism, ss469415590 (TT or ΔG), has been discovered in the region upstream IFNL3 gene, which is in high linkage disequilibrium with rs12979860. ss469415590[ΔG] is a frameshift variant that creates a novel gene, designed IFNL4, encoding the interferon-lambda 4 protein (IFNL4). The aim of the present study was to extend the analysis of IFNAR-1 mRNA levels to the ss469415590 variants. Our results highlight that the difference of IFNAR-1 mRNA levels between favourable and unfavourable genotype combinations, at both rs12979860 and ss469415590 loci, is stronger than that observed for single polymorphisms at each locus. These findings suggest may represent the biological basis for the observed association between IFNL3 CC and IFNL4 TT/TT genotypes and favourable outcome of either natural HCV infection (clearance vs chronic evolution) or IFN-based therapy.

## Introduction

The global prevalence of Hepatitis C virus (HCV) infection is estimated to be 2%, accounting for up to 180 million people infected worldwide [1]. The ability of HCV to inhibit the activation of endogenous type I interferon (IFN) system could underlie its success in establishing a chronic infection [2]. Type I IFN comprises a family of about 20 members that bind a common receptor complex, the type I IFN receptor (IFNAR) [3]. IFNs exhibit direct antiviral activity by inducing an antiviral state through expression of various intracellular genes (IFN-stimulated genes, ISGs), including IP10, whose levels are known to be prognostic factors of viral response to standard of care therapy (SOC) [4–6]. A group of recently discovered cytokines (IFN-lambda1/interleukin-29 [IL-29], IFN-lambda2/IL-28A, and IFN-lambda3/IL-28B, also designated IFNL1 to 3, respectively), assigned to a new type of IFN (type III IFN), gained increased attention in the HCV field [7]. The biological activity of type III IFN overlaps to some extent with that of type I IFN, and similar subsets of ISGs are induced as well [8]. However, there are important kinetic differences in the induction of ISGs by type I and type III IFN, and these differences may indeed produce distinct functional activities [9].

Genome-wide association studies (GWAS) identified several single-nucleotide polymorphisms (SNPs) in *IFNL3* genomic region that are strongly related to spontaneous and therapy-induced HCV clearance rate [10–13]. Numerous investigators confirmed the importance of *IFNL3* SNPs in the therapeutic response of HCV patients to SOC [14,15]. Among the identified SNPs, rs12979860 appeared the most relevant, being the rs12979860-favorable CC genotype associated with a more than two-fold increased rate of sustained virologic response (SVR) with respect to hapless (CT or TT) genotypes [16]. More recently, a new transiently induced gene near the *IFNL3* gene has been discovered, harbouring a dinucleotide variant ss469415590 (TT or ΔG), which is in high linkage disequilibrium with rs12979860. The ss469415590 [ΔG] allele is a frameshift variant that creates a novel gene, *IFNL4*, encoding a fully functional protein designated interferon-lambda 4 (IFNL4) [17,18]. The ss469415590 [TT] allele, which abrogates the production of the IFNL4 protein, is considered the favourable haplotype towards response to SOC, while the ss469415590 [ΔG] is considered the unfavourable one [18,19].

The biological basis for the influence of *IFNL3* polymorphisms on HCV infection is not clear so far, and is probably complex, involving several host functions, exerted both at tissue (liver) and systemic (i.e. blood) level [20]. To this respect, peripheral blood mononuclear cells (PBMC) are important players in the struggle against HCV [21–23], and, although not considered as main target of HCV replication, their functions are impaired by indirect mechanisms involving HCV-induced cytokines or viral products [24–27]. It has been shown that enhanced expression of ISGs, not only in the liver, but also in PBMC, is a negative prognostic factor of virological response to IFN therapy [5, 28–30].

Previous findings from our group showed that IFNAR-1 mRNA levels are strongly reduced in HCV-infected subjects, and the reduction is much stronger in individuals with rs12979860 CT/TT alleles as compared to CC [31]. We hypothesized that endogenously produced IFN-lambda may be responsible for the partial reversal of the impairment IFNAR-1 expression in CC individuals, possibly conferring to these individuals a response advantage to either endogenous or exogenous IFN-alpha [31].

In the present study the analysis of IFNAR-1 mRNA levels was extended to the ss469415590 variants, either alone or in conjunction with the rs12979860 polymorphisms.

## Materials and Methods

The project was approved by the Local Ethical Committee, and patients agreed to participate to the study by signing informed consent.

PBMC samples from 32 treatment-naїve patients, chronically infected with HCV (genotype 1 or 4), collected at the National Institute for Infectious Diseases and stored in the Institutional Biorepository, for whom the rs12979860 (here designated *IFNL3*) and ss469415590 (here designated *IFNL4*) genotypes were known, were retrospectively selected to perform a comparative analysis.

*IFNL3* rs12979860 genotype was established on genomic DNA, using a custom made TaqMan assay previously described [31]. *IFNL4* ss469415590 genotype was established using a method established by Prokunina-Olsson and collaborators [18]. Allelic discrimination was achieved using the SDS 1.3 software [18].

Total cellular RNA was extracted from PBMC using Trizol (Gibco BRL, Grand Island, NY, USA) and reverse-transcribed by TaqMan Reverse Transcription Reagent kit (Applied Biosystems, Foster City, CA, USA). The quantification of IFNAR-1 and IP10 mRNA was performed by Taqman real-time RT-PCR methods previously established in our laboratory [5,32]. Results were expressed as ratio to beta-actin.

In a set of experiments the PBMC were exposed for 3h to either medium or 10^3^ IU/ml recombinant human IFN-alpha2b (Intron; Schering Corp., Kenilworth, NJ, USA; specific activity: 400MIU/mg,1IU corresponding to 2.5pg). The timing and dose of IFN–alpha experiment was selected on the basis of a preliminary experiment performed on healthy donor PBMC. In fact, the 3h time point corresponds to peak of IP10 mRNA, and at this time point the enhancement is at plateau from 10^2^ IU/ml onward. However, we selected 10^3^ IU/ml to avoid the individual variability due to the possible suboptimal induction at lower doses.

Statistical analyses were performed by Prism 4 software (GraphPad, San Diego, CA). Differences were evaluated by the non parametric Mann-Whitney U test or by Student’s t test, as appropriate. Correlations were analyzed by Pearson r test. Differences with p<0.05 were considered statistically significant.

## Results

**Fig 1:**
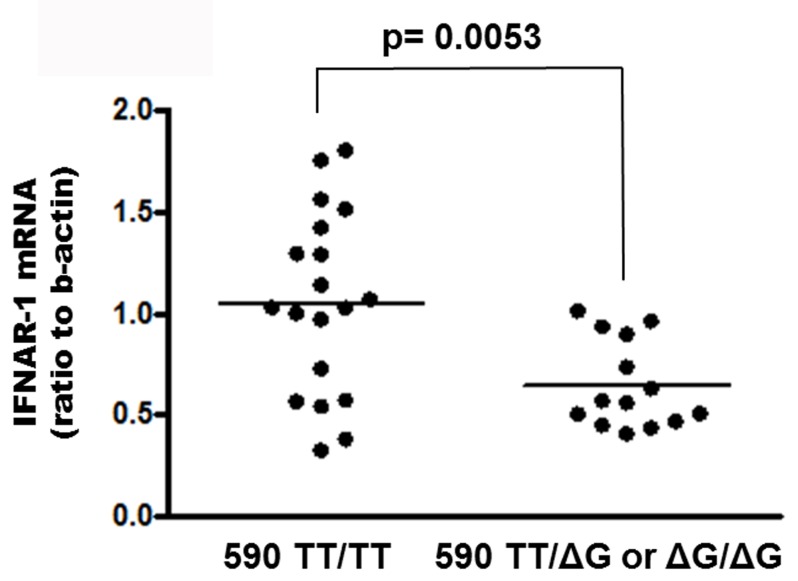
Levels of IFNAR-1 mRNA in PBMC from HCV-infected naїve subjects carrying *IFNL4* ss469415590 TT/TT genotype vs patients carrying the ΔG allele. Results are expressed as ratio to beta-actin. The horizontal bar indicates median.

The distribution of *IFNL3* genotypes was: 8 CC (25%), 13 CT (40.5%), 11 TT (34.5%); the distribution of *IFNL4* genotypes was: 18 TT/TT (56.2%), 10 TT/ΔG (31.3%), 4 ΔG/ΔG (12.5%) (Table 1). As shown in Fig. 1, median levels of IFNAR-1 mRNA in PBMC from patients carrying *IFNL4* TT/TT were 1,82 fold higher than those from patients carrying the ΔG allele (either TT/ΔG or ΔG/ΔG) [median: 1.026 (IQR: 0.5710–1.420) vs 0.5640 (0.4585–0.9135) p = 0.0053]. No correlation between IFNAR-1 expression and HCV viral load was observed (r = -0.1152; p = 0.6598).

**Table 1:** Characteristics of 32 treatment naive HCV-infected patients included in the study.

Age [median (range)] years	53.5 (30–81)
Sex (Male, Female)	25, 7
AST [median (range)] IU/L	41.5 (17–162)
ALT [median (range)] IU/L	63 (13–310)
γ-GT [median (range)] IU/L	38 (9–599)
HCV load [median (range)] Log_10_ IU/ml	6.12 (4.22–6.84)
HCV genotypes (gt1, gt4)	22, 10
IFNL3 rs 12979860 genotypes (CC, CT, TT)	8, 13, 11
IFNL4 ss469415590 genotypes (TT/TT, TT/ΔG, ΔG/ΔG)	18,10, 4

When considering the patients grouped according to the *IFNL3* and *IFNL4* genotype combinations, 8 patients were *IFNL3* CC and *IFNL4* TT/TT (*IFNL3* and *IFNL4* favourable), Group 1; 11 patients were *IFNL3* CT or TT and *IFNL4* TT/TT (*IFNL3* unfavourable and *IFNL4* favourable), Group 2; 14 patients were *IFNL3* CT or TT and *IFNL4* TT/ΔG or ΔG/ΔG (*IFNL3* and *IFNL4* unfavourable), Group 3.

**Fig 2:**
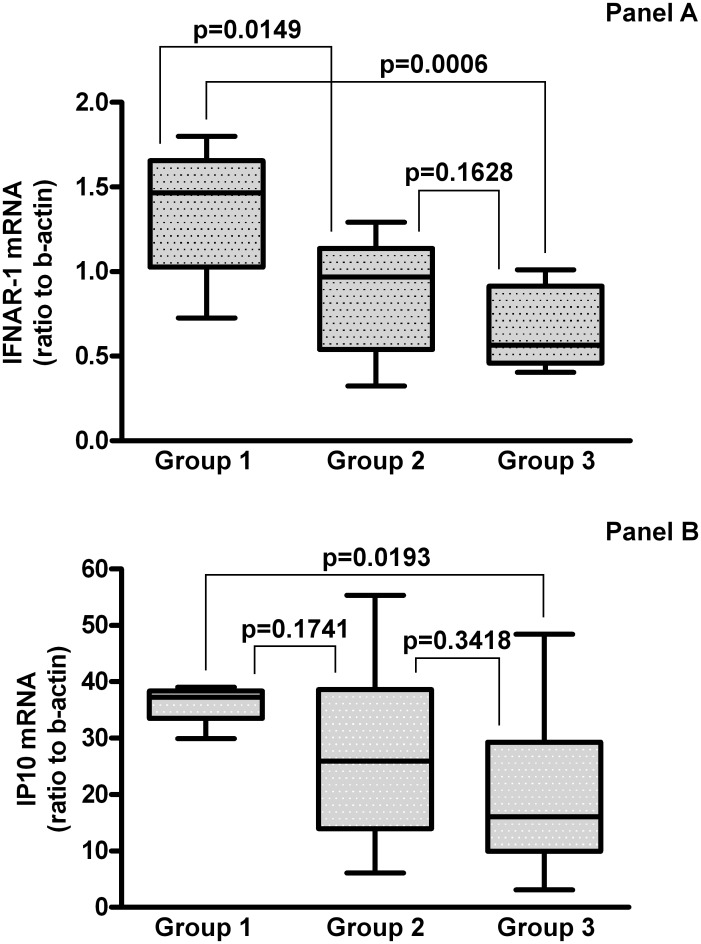
Levels of IFNAR-1 mRNA in PBMC from the HCV-infected naïve subjects grouped according to their *IFNL3* and *IFNL4* genotype combinations. Panel A. Group 1: *IFNL3* CC and *IFNL4* TT/TT (*IFNL3* favourable, *IFNL4* favourable), n = 8; Group 2: *IFNL3* CT or TT and *IFNL4* TT/TT (*IFNL3* unfavourable and *IFNL4* favourable) n = 10; Group 3: *IFNL3* CT or TT and *IFNL4* TT/ΔG or ΔG/ΔG (*IFNL3* and *IFNL4* unfavourable) n = 14. The results are expressed as ratio to beta-actin (median, IQR). Levels of IP10 mRNA in PBMC from the various groups after 3h of exposure to 10^3^ IU/ml IFN-alpha2b *in vitro*, according to their *IFNL3* and *IFNL4* genotype combinations. Panel B. Group 1: *IFNL3* CC and *IFNL4* TT/TT (*IFNL3* favourable, *IFNL4* favourable) n = 6; Group 2: *IFNL3* CT or TT and *IFNL4* TT/TT (IFNL3 unfavourable and *IFNL4* favourable) n = 11; Group 3: *IFNL3* CT or TT and *IFNL4* TT/ΔG or ΔG/ΔG (*IFNL3* and *IFNL4* unfavourable) n = 11. The results are expressed as ratio to beta-actin, after subtraction of values from unexposed cultures (median, IQR). The range of IP10 mRNA levels in unexposed PBMC cultures was 0,375 to 0,967.

The median levels of IFNAR-1 mRNA in PBMC from patients with the various *IFNL3* and *IFNL4* genotype combinations are shown in Fig. 2 Panel A. As can be seen (Fig. 2 Panel A) PBMC from patients with favourable combination at both *IFNL3* and *IFNL4* loci contain higher levels of IFNAR-1 mRNA as compared to the other groups. In particular, the most prominent difference (2.6 fold) was observed for Group 1 vs Group 3 [median: 1.466 (IQR: 1.025–1.655) vs 0.564 (IQR: 0.458–0.913); p = 0.0006]; an 1.51 fold difference was observed vs Group 2 [median:0.969 (IQR: 0.540–1.137); p = 0.0149]; the difference between Group 2 and Group 3 was not statistically significant [p = 0.1628].

**Fig 3:**
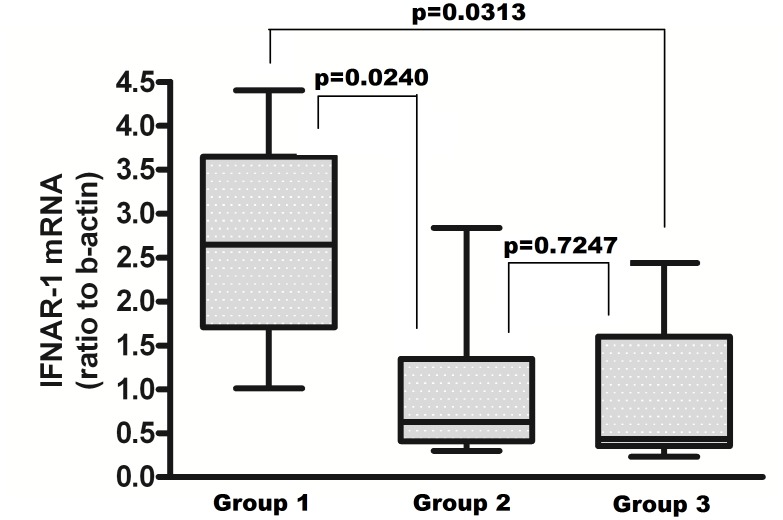
Levels of IFNAR-1 mRNA in PBMC from the HCV-infected naïve subjects after 3h of exposure to 10^3^ IU/ml IFN-alpha2b *in vitro*, according to their *IFNL3* and *IFNL4* genotype combinations. Group 1: *IFNL3* CC and *IFNL4* TT/TT (*IFNL3* favourable, *IFNL4* favourable) n = 6; Group 2: *IFNL3* CT or TT and *IFNL4* TT/TT (*IFNL3* unfavourable and *IFNL4* favourable) n = 11; Group 3: *IFNL3* CT or TT and *IFNL4* TT/ΔG or ΔG/ΔG (*IFNL3* and *IFNL4* unfavourable) n = 11. The results are expressed as ratio to beta-actin, after subtraction of values from unexposed cultures (median, IQR).

To explore whether the differences in the levels of IFNAR-1 mRNA could lead to an increased response to exogenously administered IFN-alpha, PBMC from patients with the various genotype combinations were exposed to IFN-alpha and the induction of mRNA for IP10, as biomarker of IFN activity, was measured. The results, shown in Fig. 2 Panel B, indicate a gradient of IFN response among the groups in term of IP10 mRNA induction, that paralleled the levels of IFNAR-1 expressed before IFN-alpha treatment (Fig. 2 Panel A). In particular, the difference between Group 1 and Group 3 was highly significant [median: 37.28 (IQR: 33.53–38.37) vs 16.06 (IQR: 9.979–29.27); p = 0.0193]. IFNAR-1 mRNA levels were not significantly modified by IFN-alpha treatment (Fig. 3), reflecting the differences among groups observed at baseline.

## Discussion

Several studies confirmed the importance of *IFNL3* SNPs in the kinetics of HCV RNA decay and, ultimately, in the response to PEG-IFN plus RBV treatment [14,15]. In addition, *IFNL3* genotype has been correlated with the expression of IFN-lambda receptor-1 and with non-responsiveness to IFN-alpha therapy [33].

Recently, a polymorphism within the gene encoding IFNL4 protein (ss469415590 TT or ΔG), in strong linkage disequilibrium with rs12979860 polymorphism, has been described, being more strongly associated with HCV clearance and treatment outcome than the previous one [18,19].

More recently, *IFNL4* genotype has also been shown to be correlated with the probability of virus eradication in patients receiving IFN-free therapy [34]. Based on these evidences and considering a previous study in which our group observed higher levels of IFNAR-1 mRNA in PBMC from patients with favourable *IFNL3* genotype [31], we extended the analysis to *IFNL4* polymorphisms, considering also the combination of alleles at both *IFNL3* and *IFNL4* loci.

Our results highlighted a significantly higher expression (1.82 fold) of IFNAR-1 mRNA in PBMC from patients carrying *IFNL4* TT/TT genotype vs patients carrying the ΔG allele. More interestingly, the most prominent difference of IFNAR-1 mRNA levels was observed between patients with the most favourable combination at both *IFNL3* and *IFNL4* loci with respect to patients with combinations involving any unfavourable allele. Overall, the difference between the favourable and unfavourable combinations at both loci was much stronger than that observed for the single polymorphisms at each locus, i.e. 2.6 fold for the combination vs 1.82 fold for *IFNL4* (present study) and 2.3 fold for *IFNL3* [31].

The higher expression of IFNAR-1 in the favourable combination is biologically relevant, since it is connected with a stronger response to IFN-alpha exposure *in vitro*, in terms of IP10 mRNA induction.

One limitation of this study is that we could not evaluate any correlation between *IFNL3* and *IFNL4* genotypes, baseline levels of IFNAR-1 and therapeutic response, since only few patients included in this study underwent IFN-based treatment so far (12 overall, 4 SVR and 8 non SVR).

The findings from the present study, if confirmed in larger studies and supported by treatment outcome data, may offer a key for elucidating the biological basis of the advantage represented by *IFNL3* and *IFNL4* favourable genotypes towards natural or therapy-induced HCV clearance.
